# Addendum: Telomouse—a mouse model with human-length telomeres generated by a single amino acid change in RTEL1

**DOI:** 10.1038/s41467-026-71821-1

**Published:** 2026-05-01

**Authors:** Riham Smoom, Catherine Lee May, Vivian Ortiz, Mark Tigue, Hannah M. Kolev, Melissa Rowe, Yitzhak Reizel, Ashleigh Morgan, Nachshon Egyes, Dan Lichtental, Emmanuel Skordalakes, Klaus H. Kaestner, Yehuda Tzfati

**Affiliations:** 1 Department of Genetics, The Silberman Institute of Life Sciences, Safra Campus, The Hebrew University of Jerusalem, Jerusalem, 91904 Israel; 2 Department of Genetics and Institute for Diabetes, Obesity and Metabolism, Perelman School of Medicine, University of Pennsylvania, Philadelphia, PA 19104 USA; 3 Division of Gastroenterology and Hepatology, Department of Medicine, Hospital of the University of Pennsylvania, Philadelphia, PA 19104 USA; 4 Department of Pharmacology and Toxicology, Massey Cancer Center, Virginia Commonwealth University, 401 College St, Richmond, VA 23298 USA; 5 Present Address: Faculty of Biotechnology and Food Engineering, Technion, Haifa, 3200003 Israel

Addendum to: *Nature Communications* 10.1038/s41467-023-42534-6, published online 23 October 2023

In this article, the authors reported an engineered mouse model, termed ‘Telomouse’, with a missense mutation in *Rtel1* (methionine 492 to a lysine; *Rtel1*^M492K^). The design of the Telomouse strain was based on the genetic association of *Rtel1* with telomere length, as identified by crossing *Mus spretus* with short telomeres to *M. musculus* with long telomeres^1^, and on the presence of the *Rtel1*^M492K^ variation in the *M. spretus Rtel1* transcripts sequences available at the time^2^. The authors hypothesized that this variation was responsible for the shorter telomeres of *M. spretus*, as compared with *M. musculus*, and predicted that a *M. musculus* strain harboring this mutation would also shorten its telomeres. While Telomice homozygous for the *Rtel1*^M492K^ mutation indeed shortened their telomeres as predicted, the more recently available *M. spretus* genome^3^ revealed no such variation in *Rtel1*. Furthermore, the authors obtained DNA samples from two available *M. spretus* strains^4,5^, PCR-sequenced this part of *Rtel1*, and found that both strains have a methionine at position 492. *M. spretus* appears highly heterogenous^6^ and the authors cannot exclude the possibility that the original strain used for the cDNA sequencing indeed had a lysine at position 492. However, since two currently used *M. spretus* strains have short telomeres but not the *Rtel1*^M492K^ variation, such a variation cannot explain the short telomeres in this species. Importantly, introducing *Rtel1*^M492K^ mutation into *M. musculus* resulted in short, stable, human-length telomeres in an invaluable model for studying the roles of telomeres in cancer and aging.
