# Supplementary material for: Telomouse—a mouse model with human-length telomeres generated by a single amino acid change in RTEL1
Source: Nat Commun. 2023 Oct 23;14:6708. doi: 10.1038/s41467-023-42534-6 (PMC10593777; doi:10.1038/s41467-023-42534-6)
Supplement: Supplementary file 1 — Supplementary Information [file 41467_2023_42534_MOESM1_ESM.pdf]

Supplementary Figures

Telomouse – a mouse model with human-length telomeres generated by a single amino acid change in RTEL1

Riham Smoom[1], Catherine Lee May[2], Vivian Ortiz[2,4], Mark Tigue[2], Hannah M. Kolev[2], Melissa Rowe[2], Yitzhak Reizel[2,5], Ashleigh Morgan[2], Nachshon Egyes[1], Dan Lichtental[1], Emmanuel Skordalakes[3], Klaus H. Kaestner[2*] and Yehuda Tzfati[1*]

[1]Department of Genetics, The Silberman Institute of Life Sciences, Safra Campus, The Hebrew University of Jerusalem, Jerusalem, 91904, Israel

[2]Department of Genetics and Institute for Diabetes, Obesity and Metabolism, Perelman School of Medicine, University of Pennsylvania, Philadelphia, PA 19104, USA

[3]Department of Pharmacology and Toxicology, Massey Cancer Center, Virginia Commonwealth University, 401 College St, Richmond, VA 23298-0035, USA

[4]Division of Gastroenterology and Hepatology, Department of Medicine, Hospital of the University of Pennsylvania, Philadelphia, PA 19104, USA

[5]Present address: Faculty of Biotechnology and Food Engineering, Technion, Haifa, Israel, 3200003

[*]Correspondence: tzfati@mail.huji.ac.il (Y.T.) and kaestner@pennmedicine.upenn.edu (K.H.K.)

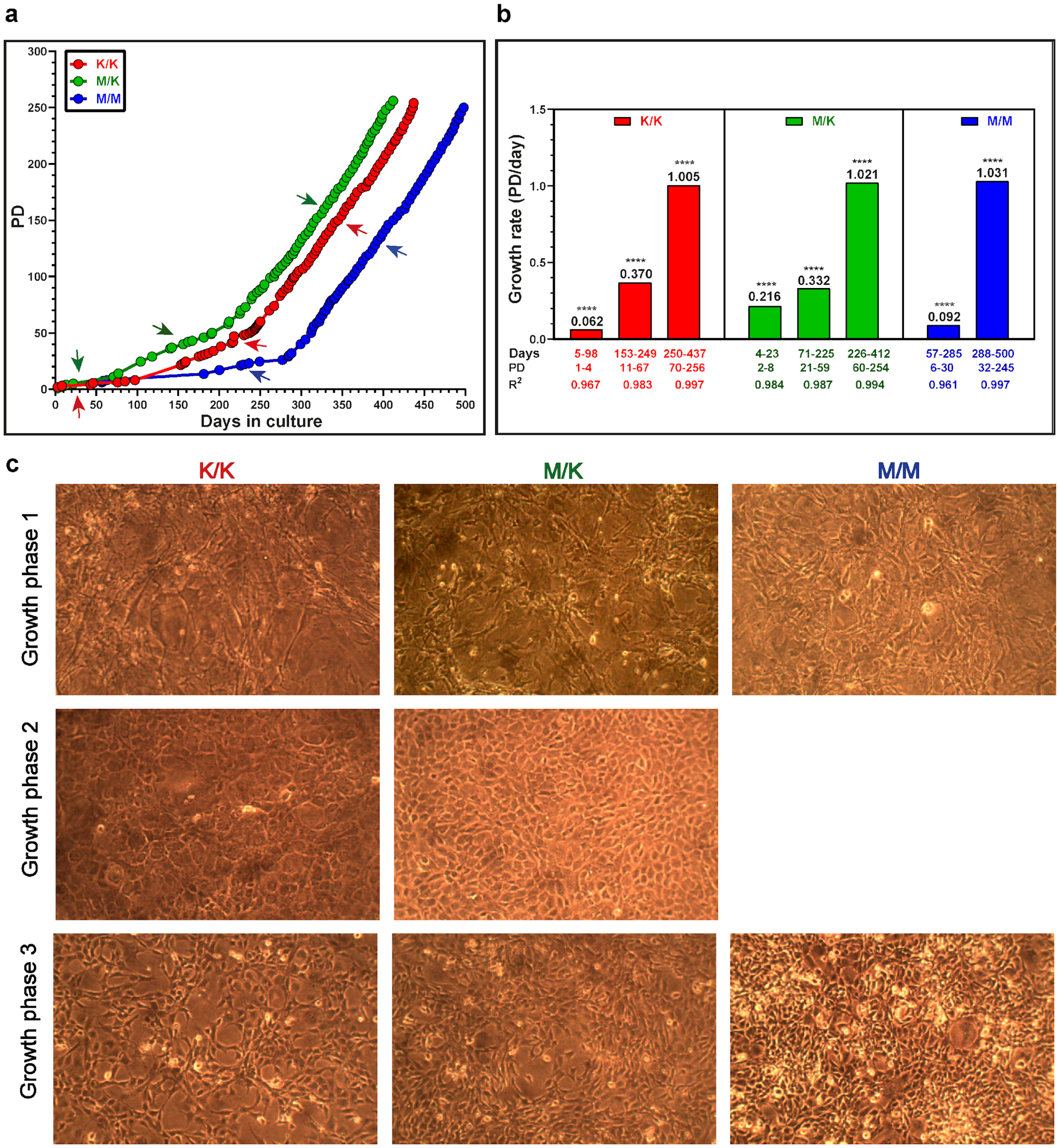

Figure S1. Immortalization and growth characteristics of MEFs from WT and *Rtel1*^{M492K} mutant mice. (a) MEF cultures were prepared from F3 littermate embryos (K/K and M/K) and from WT *Mus musculus* embryos (M/M), immortalized by serial passaging and grown to PD 250. Arrows indicate different growth phases. (b) Growth rates were calculated based on the graphs in (a) and indicated above the bars. Days, PD, and coefficient of determination (R^2), for the linear regression line calculated for each growth phase are indicated below. **** P < 0.0001. (c) Representative images show different cell morphology for each growth phase.

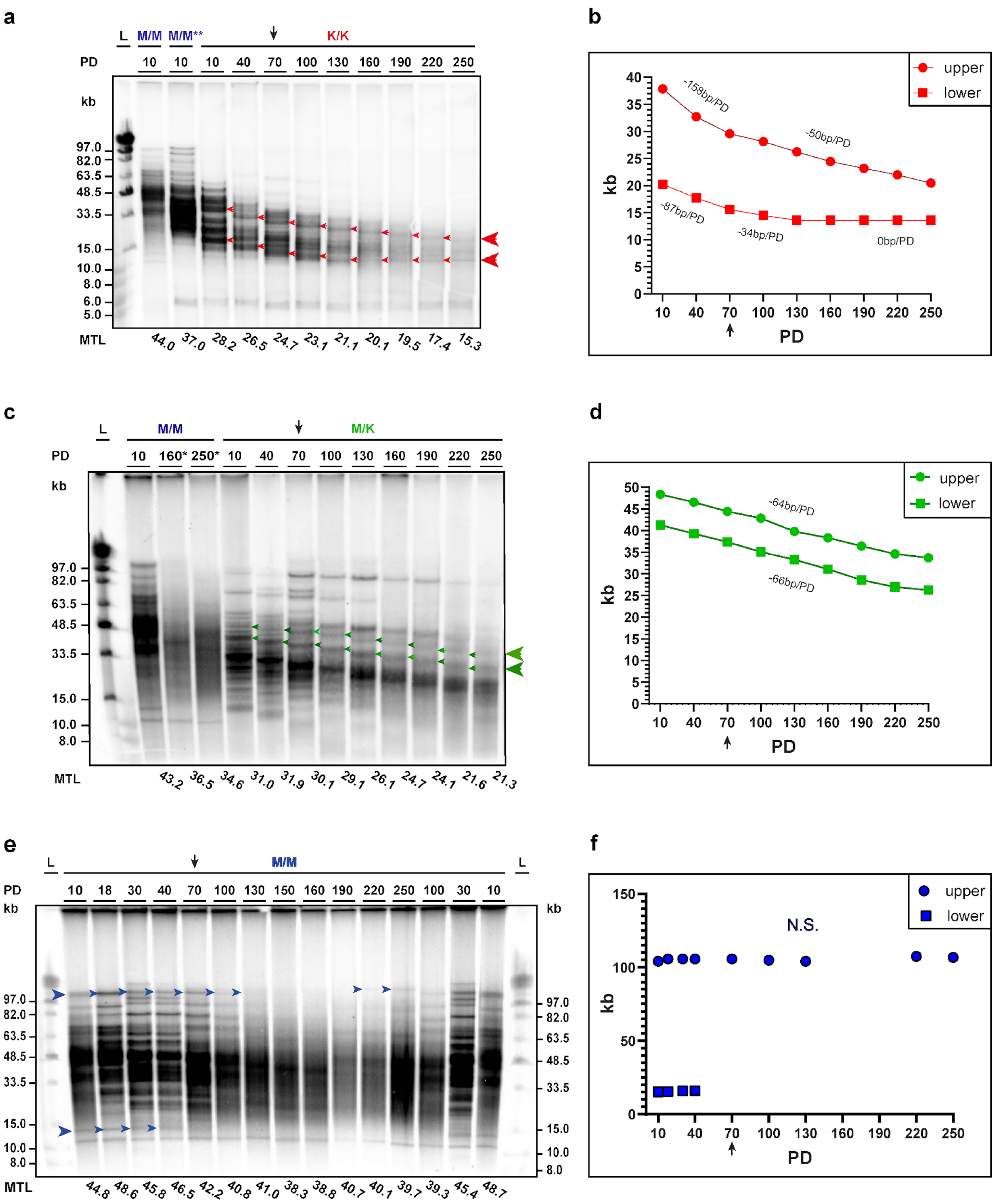

Figure S2. MEF telomeres display a distinct banding pattern. (a, c, e) Genomic DNA samples prepared from *Rtel1*^{M492K} homozygous (K/K) or heterozygous (M/K) mutant or WT (M/M) MEF cultures were analyzed by PFGE and in-gel hybridization. The change of cell morphology (see Figure S1c), reflecting cellular immortalization, is indicated by vertical arrows above the PD 70 lanes. M/M** represents a MEF culture derived from a progeny of two heterozygous RTEL1 mutant mice. Since this embryo inherited short telomeres it is not considered as 'true' WT and thus was excluded from further analysis. M/M PD 160* and 250* DNA samples were suspected to be degraded based on PFGE of uncut DNA, and were also excluded. (b, d, f) The length of distinct TRF bands indicated by small arrows on the gel images was calculated and plotted. The rate of shortening is indicated. 'N.S.', no significant deviation from a horizontal line with a slope of 0.

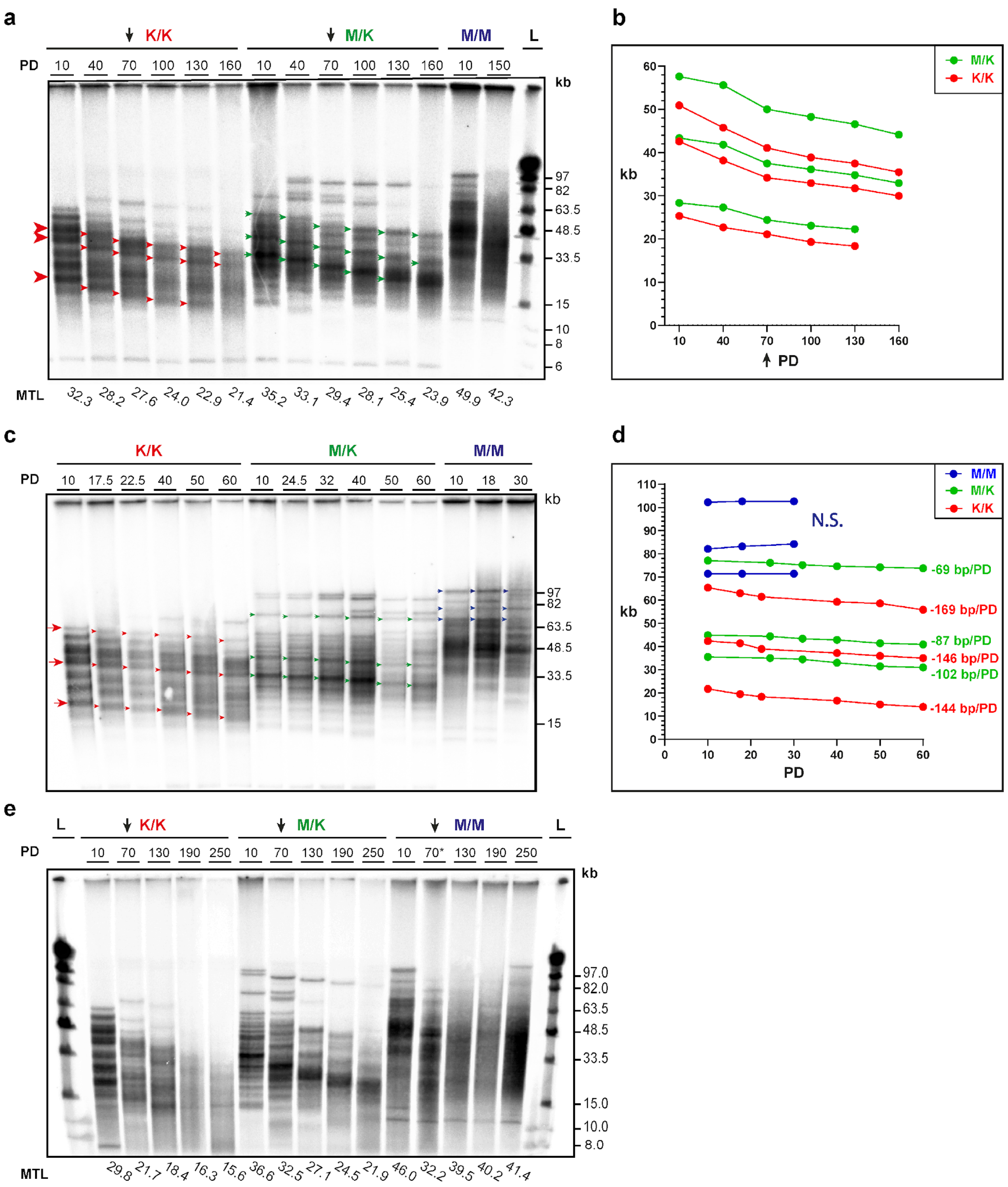

Figure S3. MEF telomeres display a distinct banding pattern. (a, c, e) Genomic DNA samples prepared from *Rtel1*^{M492K} homozygous (K/K), heterozygous (M/K), or WT (M/M) MEF cultures were analyzed by PFGE and in-gel hybridization. The change of cell morphology (see Figure S1c), reflecting cellular immortalization, is indicated by vertical arrows above the PD 70 lanes. The M/M sample PD 70* in (e) was suspected to be degraded and excluded. (b, d) The length of distinct TRF bands indicated by small arrows on the gel images was calculated and plotted. The rate of shortening is indicated. 'N.S.', no significant deviation from a horizontal line with a slope of 0.

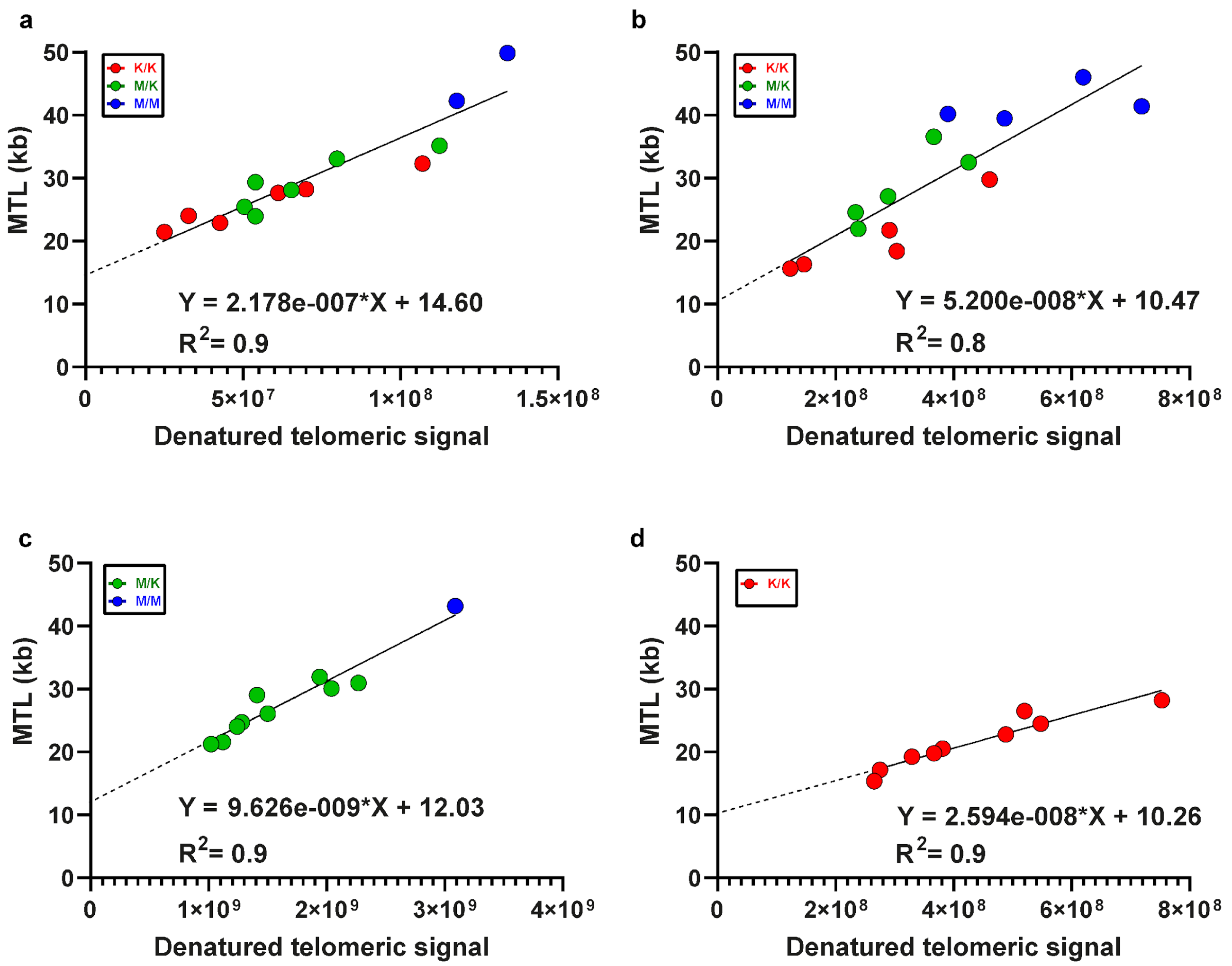

Figure S4. Telomeric repeat arrays are shorter than the telomeric restriction fragments. MTL was plotted as a function of the denatured hybridization signals quantified by *ImageQuant-TL* for the gel shown in Figure S3a (a), Figure S3e (b), Figure S2c (c), and Figure S2a (d). The linear regression line, formula and coefficient of determination (R^2) value are shown for each graph. Blue indicates M/M (WT), green M/K (heterozygous); and red indicates K/K (homozygous) samples.

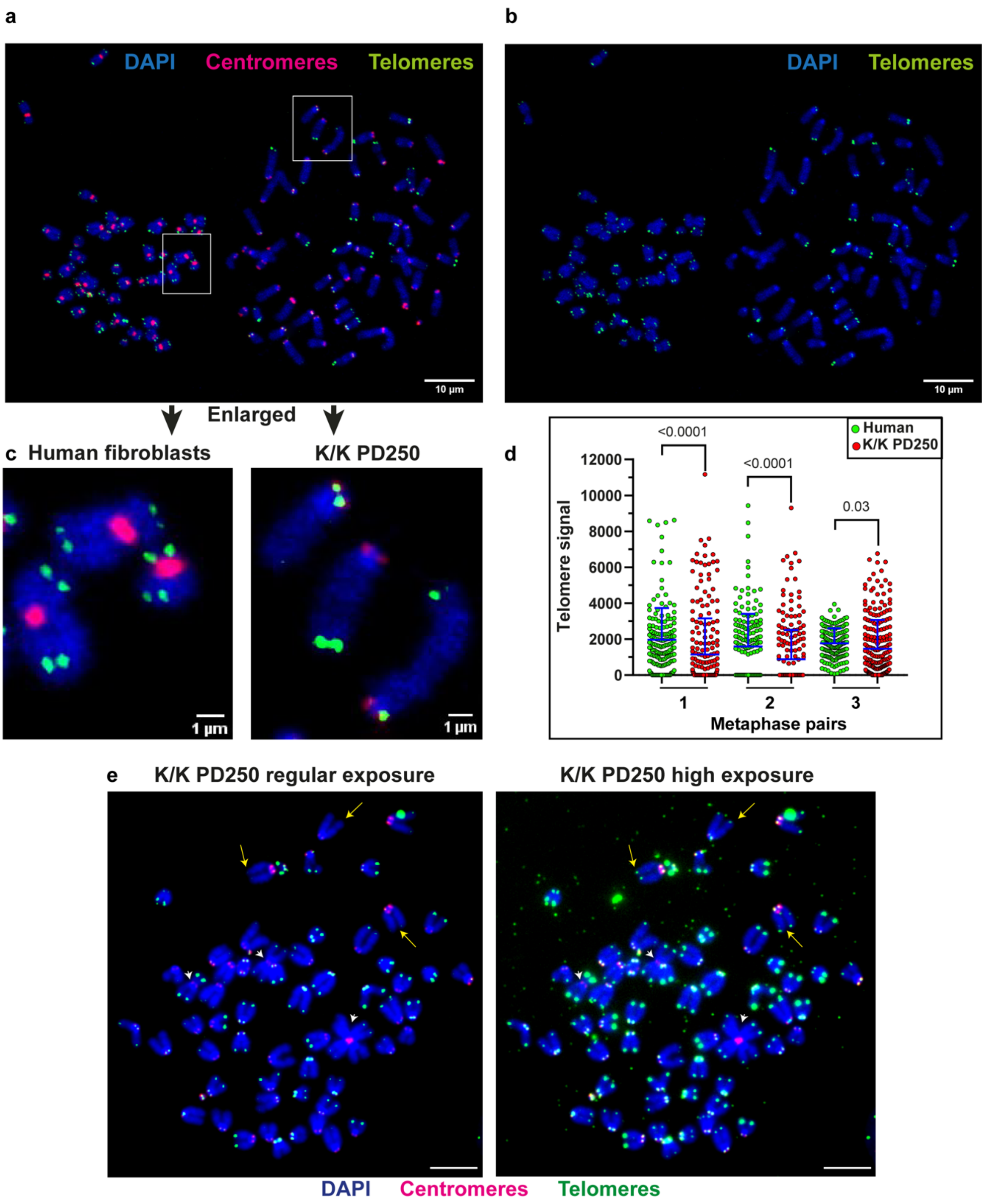

Figure S5. ***Rtel1*$^{K/K}$ MEFs telomeres are more heterogeneous than human fibroblasts telomeres.** (a) *Rtel1*$^{K/K}$ MEFs at PD 250 and human telomerase positive fibroblasts with an MTL of 14 kb (as measured by in-gel hybridization, see Figure 3d) were arrested in metaphase, mixed and spread on slides. An example of one microscope field is shown. The slides were hybridized to a green telomeric PNA probe and a red centromeric PNA probe to distinguish the mostly metacentric human chromosomes from the mouse acrocentric chromosomes. Imaging acquisition was by FV-1200 confocal microscope (Olympus, Japan). (b) Shows the same image without the centromeric signal and (c), enlargements, showing more undetected telomeric signals in K/K MEFs than human fibroblasts. (d) The telomeric signals were quantified for three images with pairs of human and mouse metaphases combined. 164 - 172 human and 236 - 244 mouse chromosome ends were quantified in each image using the *Telometer* plugin of *NIH ImageJ* and plotted. Horizontal lines indicate the mean and standard deviation. The P values were calculated by a 2-tailed unpaired t-test. (e) High exposure (right) reveals very short telomeres in KK PD 250 MEFs undetected by standard exposure (left) and qFISH (long arrows). Only telomere loss under high exposure is associated with chromosome fusion (short arrows). Scale bars equal 15 µm.

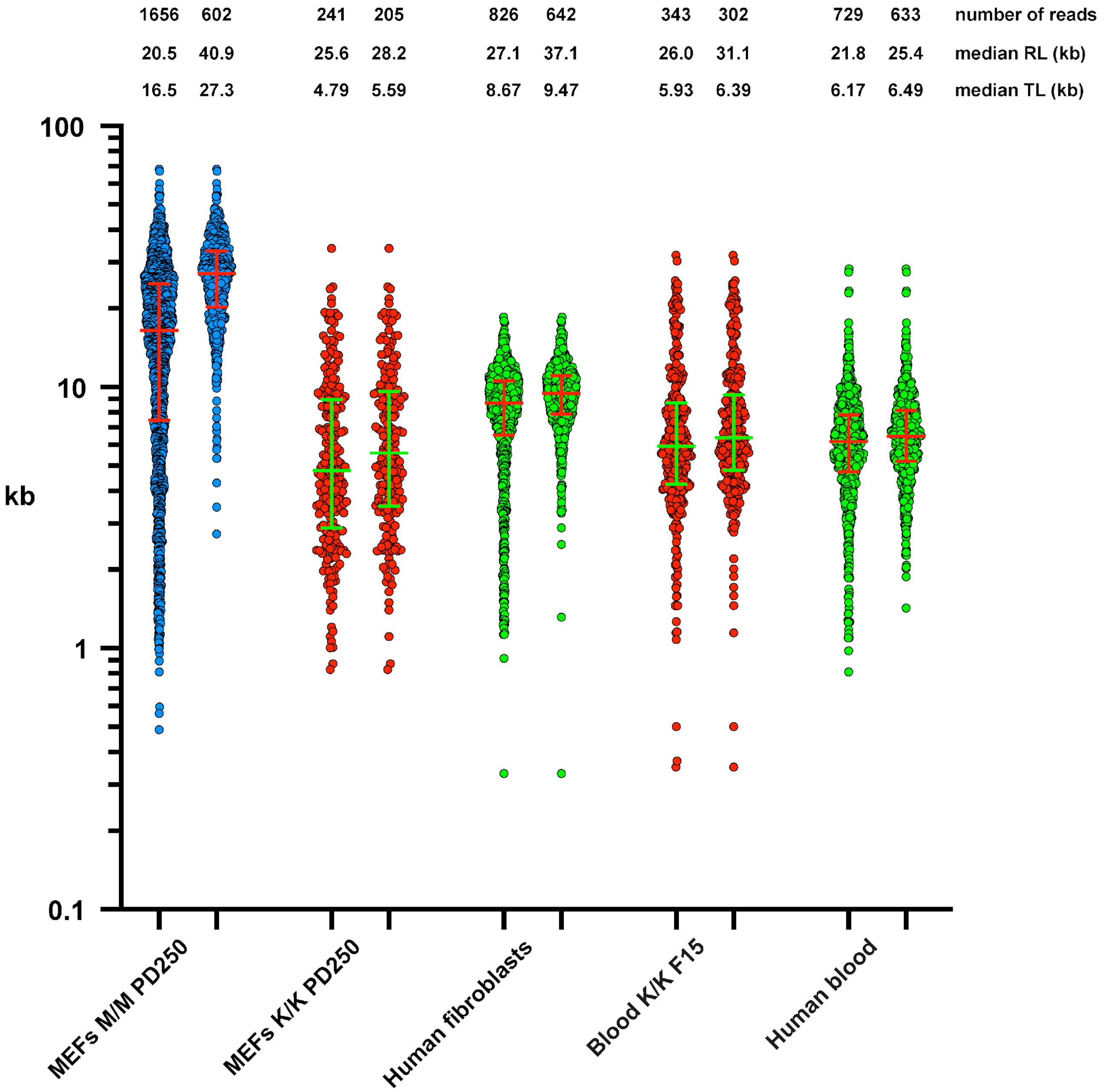

Figure S6. Filtration of telomere reads obtained by Nanopore sequencing based on read length. The length of single telomeres, as calculated by *Telomere Analyzer,* is shown on a scatter plot. The samples are the same as shown in Figures 4 and 7. For each sample, the plot on the left shows all telomeric reads and the plot on the right shows telomeres from reads longer than the corresponding running median telomere length + 100 nt (corresponding to the estimated length of the sequencing adapters; see Methods). Horizontal lines indicate the median and quartiles. The number of telomeric reads, the median read length (RL) and the median telomere length (TL) for each sample, are shown above the plots.

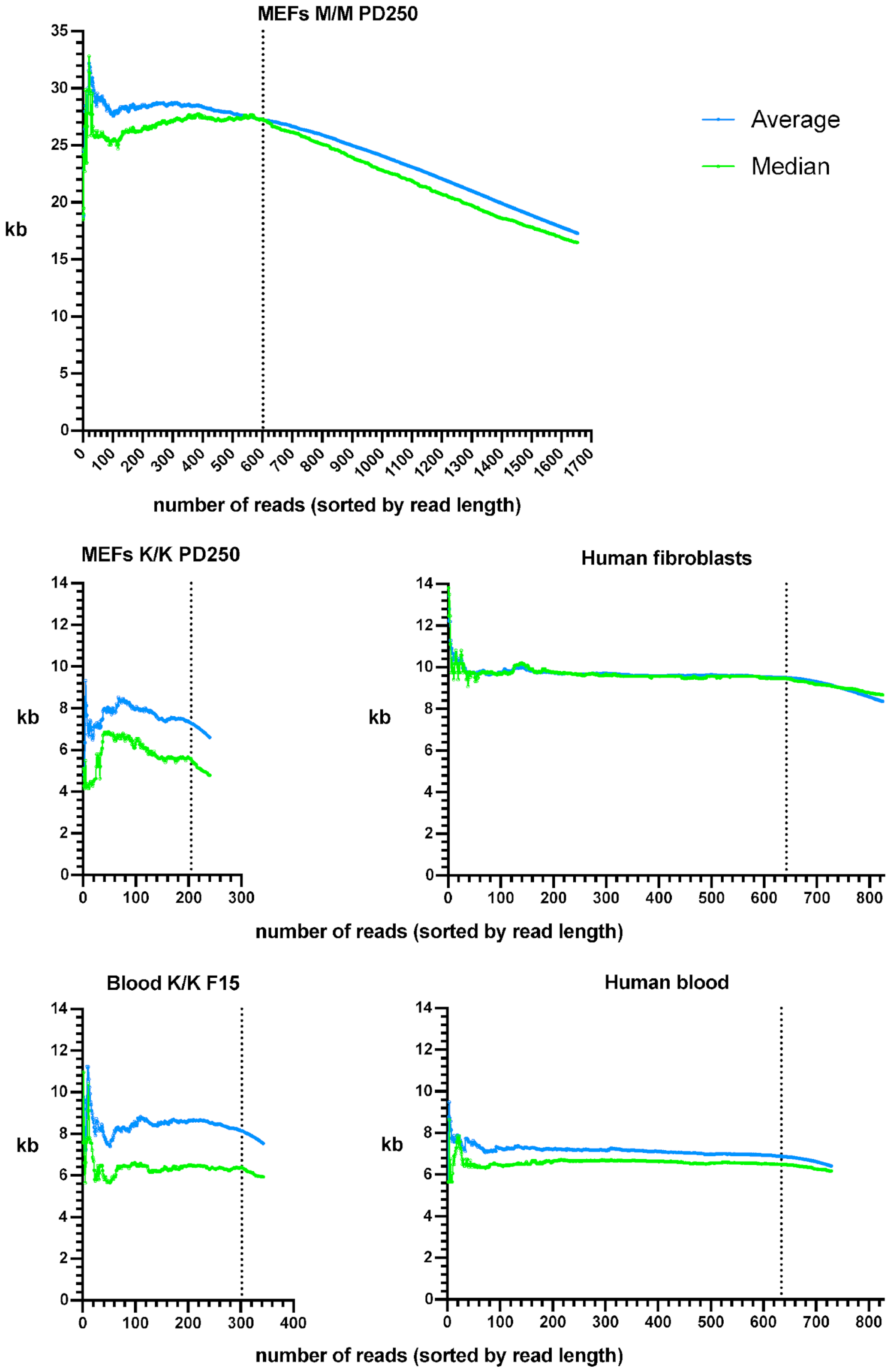

Figure S7. The influence of read length on the estimation of the median and average telomere length. The telomeric reads for each sample (same samples as in Figures 4, 7 and S6) were sorted based on their read length from largest to smallest (Supplementary Data 3). Then, 'running' median and 'running' average telomere length (TL) were calculated, meaning that for each telomere read we calculated the median and average TL of all reads of equal or greater length (Supplementary Data 3). These values were plotted in the graphs as a function of the number of included telomeric reads sorted by decreasing length. Dotted lines separate reads longer than their corresponding running median TL + 100 nt (on the left; see Methods) from shorter ones (on the right).

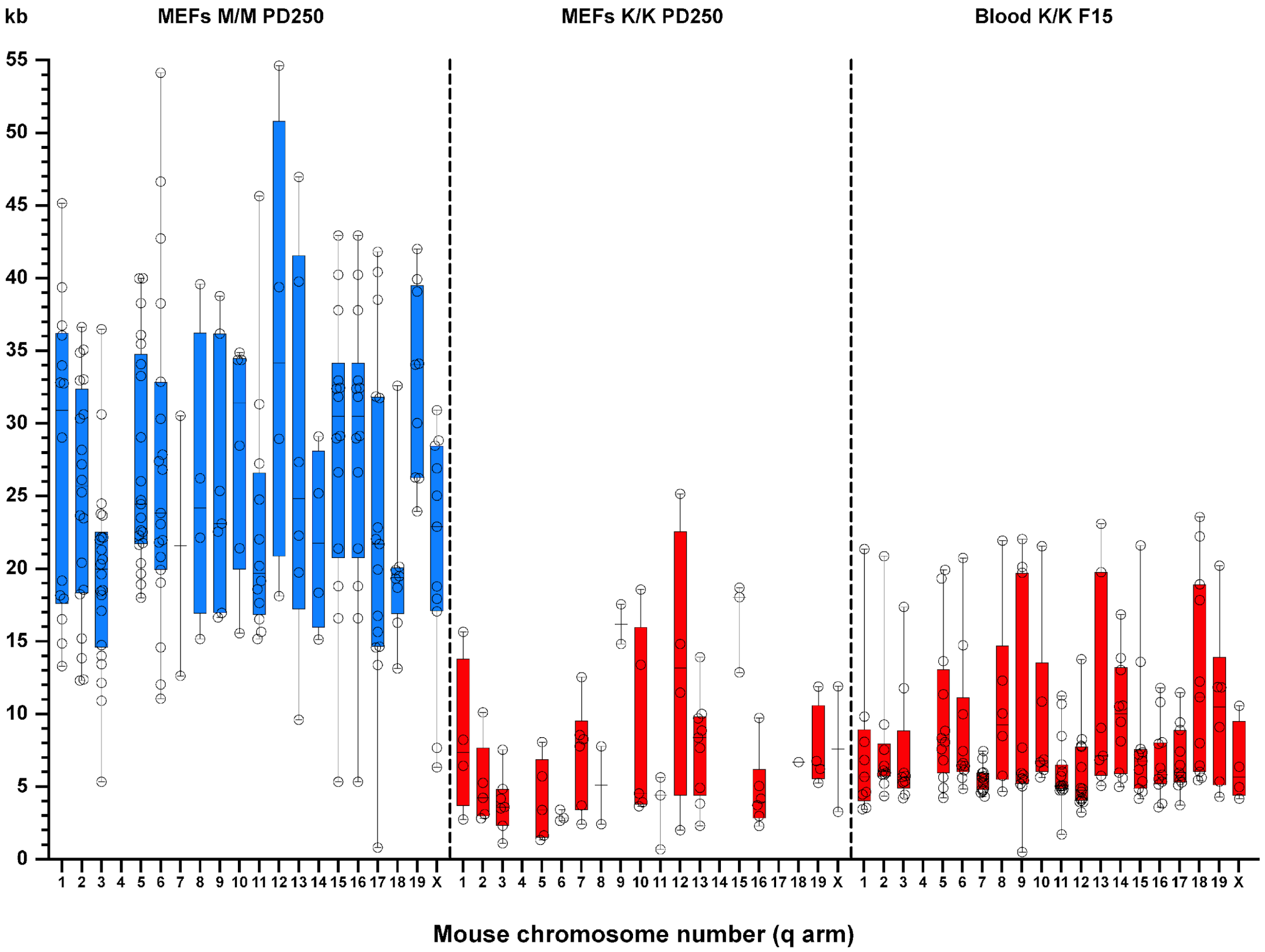

Figure S8. Mapping of telomere reads to specific mouse chromosome ends. Individual telomere reads from M/M MEFs PD250, K/K MEFs PD250, and blood from Telomice F15 (ID numbers 3045 and 2426 IDs; Supplementary Data 4) were mapped to the reference mouse genome based on the subtelomeric sequences. Open circles indicate the length of each mapped telomere. Horizontal lines indicate average telomere length, and box boundaries the quartiles.

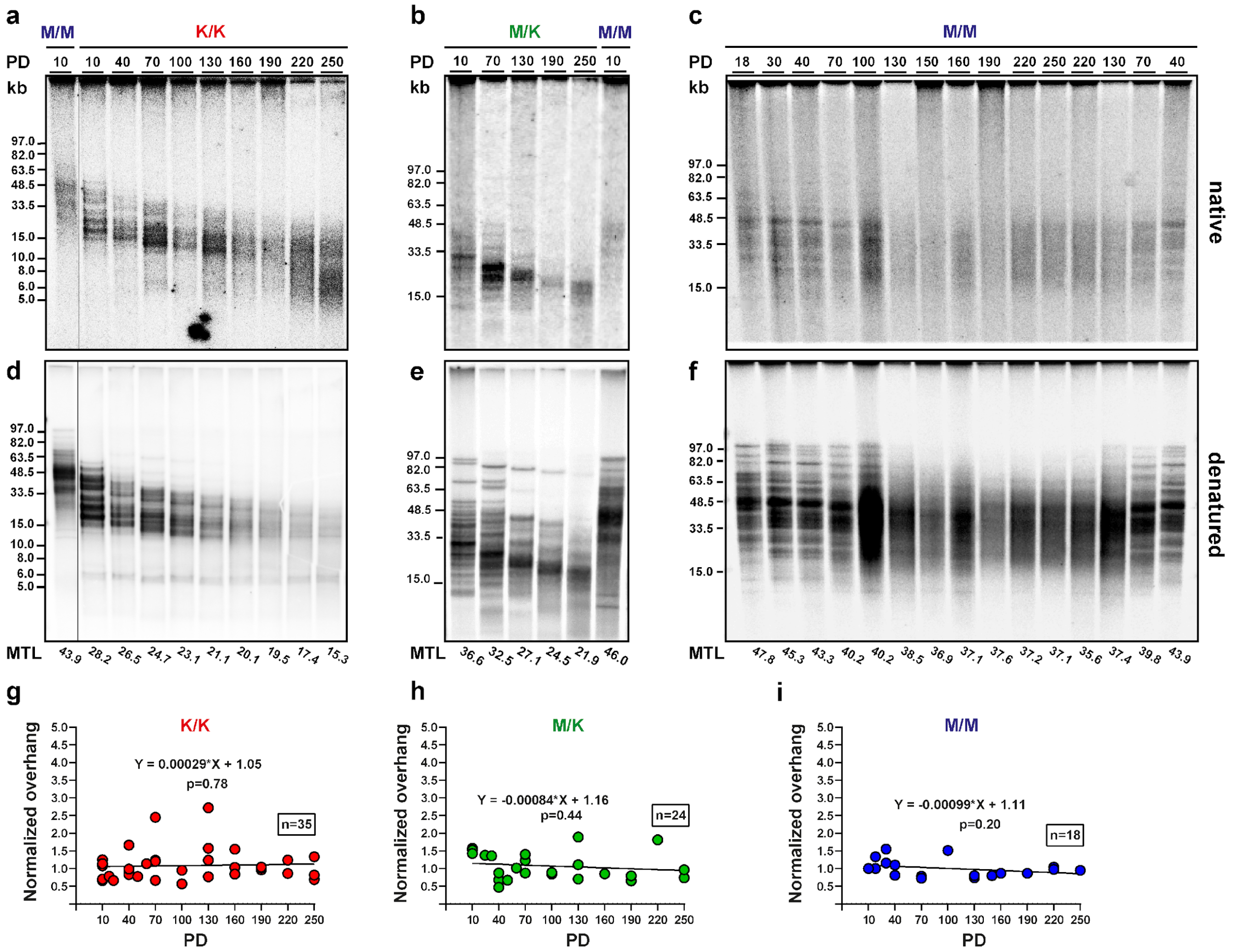

Figure S9. Normal telomeric overhang signals in *mRtel1* mutant MEFs. Equal amounts of genomic DNA from the indicated MEF cultures and PD were analyzed by PFGE and in-gel hybridization. (a, b, c) First, a telomeric C-rich probe was hybridized to the native DNA, detecting the single-stranded G-rich telomeric overhang. (d, e, f) Then, the DNA was denatured within the gel and re-hybridized to the same probe to detect the entire telomeric DNA. Representative gels are shown. (g, h, i) The native overhang signal for 77 samples in six gels was measured by *ImageQuant-TL* and plotted (summarized in Supplementary Data 1). The K/K (red) and M/K (green) samples were normalized to the same WT sample (M/M PD 10) within each gel (g, h). The M/M (blue) samples were normalized to WT sample M/M PD 18 within each gel (i). The formula for the regression line and the P value corresponding to the deviation of the slope from 0 are indicated above each graph. The high P values indicate no significant change in the overhang signal over time.

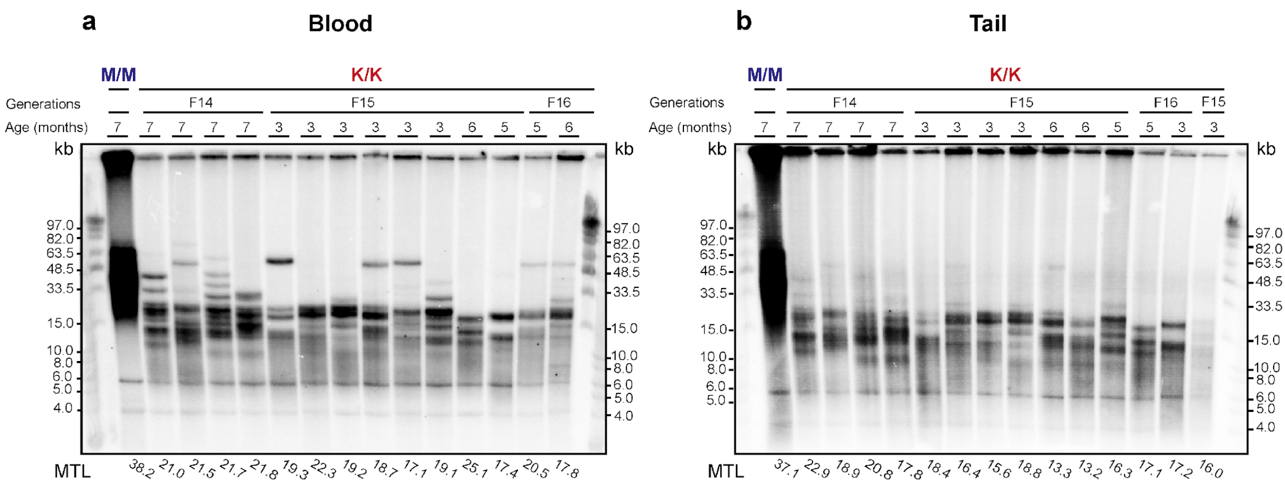

Figure S10. Telomere length stabilized at late generation Telomice. Genomic DNA samples extracted from blood leukocytes (a) or tail (b) from 14 Telomice from generations F14, F15 and F16, and one WT (M/M) mouse as a control at the indicated ages, were analyzed by PFGE and in-gel hybridization to the denatured DNA. MTL for each sample was measured by *Telotool* and indicated below the lanes.

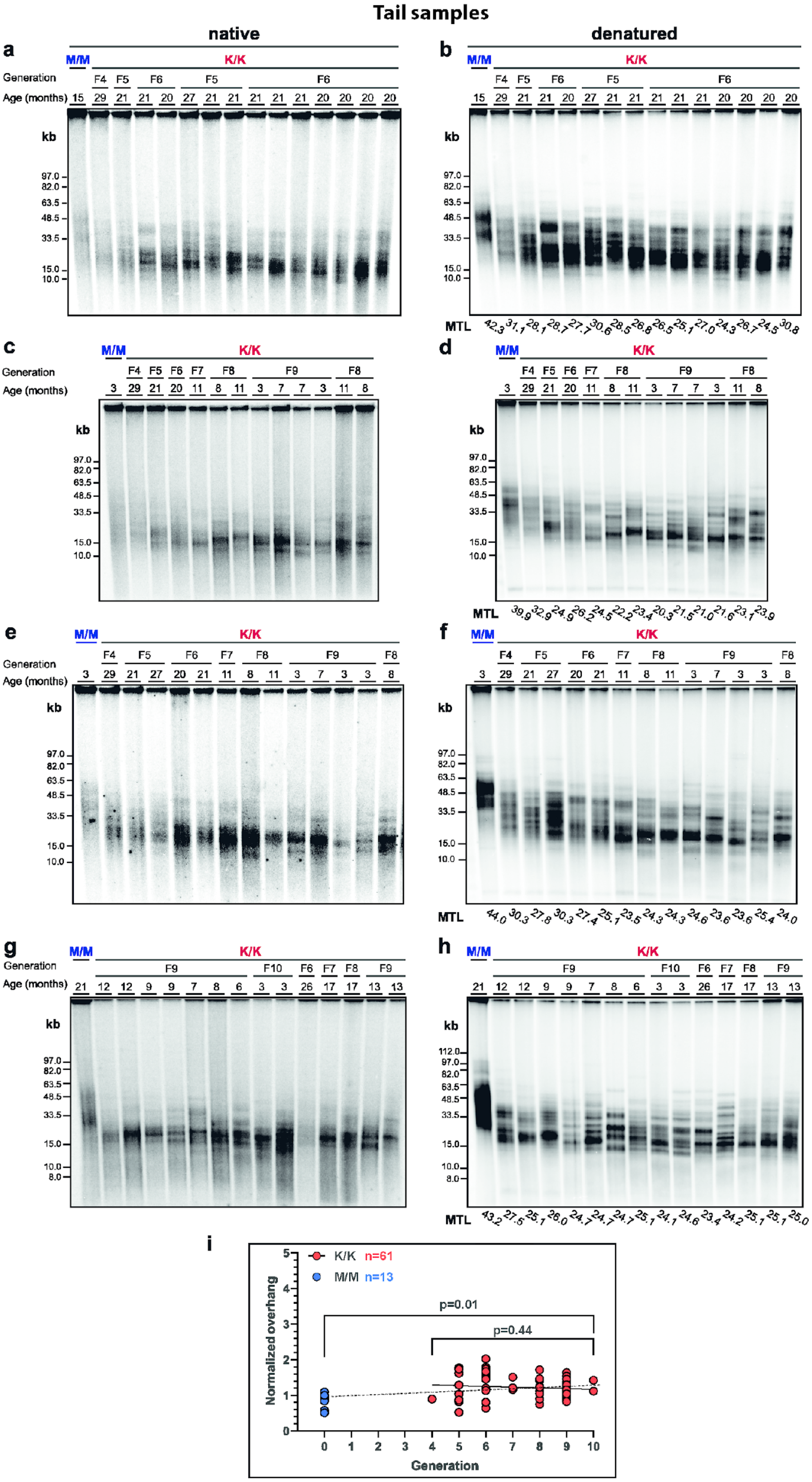

Tail samples
native
denatured
a
b
c
d
e
f
g
h
i
M/M
K/K
Generation
Age (months)
kb
MTL
42.3 31.1 28.1 28.7 27.7 30.6 28.5 26.8 26.5 25.1 27.0 24.3 26.7 24.5 30.8
39.9 32.9 24.9 26.2 24.5 22.2 23.4 20.3 21.5 21.0 21.6 23.1 23.9
44.0 30.3 27.8 30.3 27.4 25.1 23.5 24.3 24.3 24.6 23.6 23.6 25.4 24.0
43.2 27.5 25.1 26.0 24.7 24.7 24.7 25.1 24.1 24.6 23.4 24.2 25.1 25.1 25.0
K/K n=61
M/M n=13
p=0.01
p=0.44
Normalized overhang
Generation

Figure S11. No decrease in the telomeric overhang in tail samples of Telomice. (a, c, e, g) Equal amounts of genomic DNA derived from tail samples of the indicated Telomice and WT mice were analyzed by PFGE and in-gel hybridizations with a telomeric C-rich probe to the native DNA, detecting the single-stranded G-rich telomeric overhang. (b, d, f, h) The DNA was denatured within the gel and re-hybridized to the same probe to detect the entire telomeric G-strand DNA. Representative gels are shown. (i) The native signal for 61 K/K and 13 WT (M/M) tail samples were measured by *ImageQuant-TL*, and all the samples within each gel (a total of six gels) were normalized to the WT sample and plotted (summarized in Supplementary Data 4). The slope for K/K was analyzed by simple linear regression. The equation for the regression line with 95% confidence intervals was: Y= -0.020*X + 1.38, and the P value corresponding to the deviation of the slope from zero is indicated above the graph. The high P value indicates no significant change in the overhang signal while telomeres shorten over seven generations of Telomouse. Moreover, comparing the Telomice overhang signal to the WT mice, the regression line (dotted line) gave a p value of 0.01 for the equation Y= 0.0325*X + 0.97, indicating that the overhang in the Telomouse tail samples is not shorter than in the WT, and may even be longer.

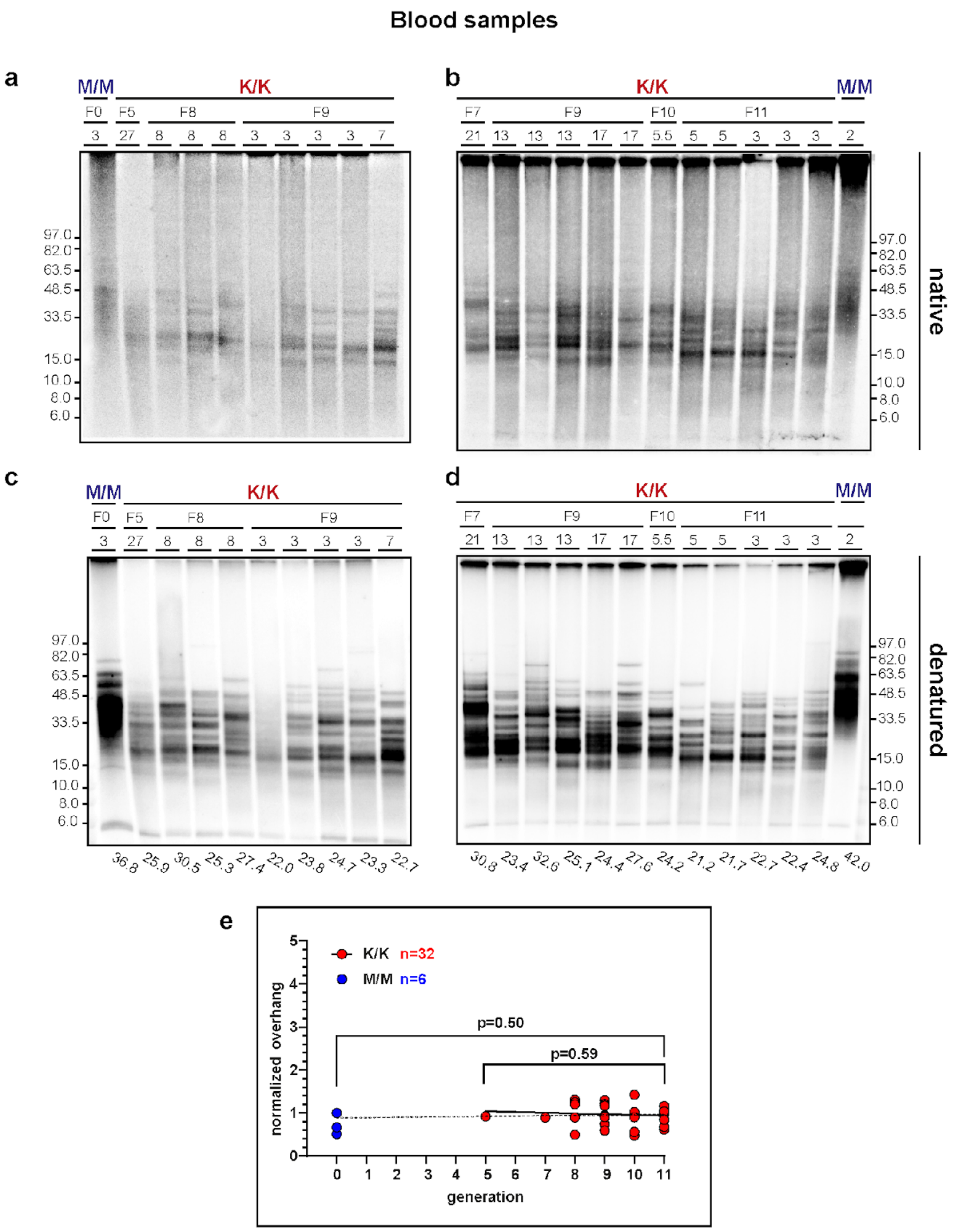

Figure S12. No decrease in the telomeric overhang signal in blood samples of Telomice. (a, b) Equal amounts of genomic DNA derived from blood samples of the indicated Telomice and WT mice were analyzed by PFGE and in-gel hybridizations with a telomeric C-rich probe to the native DNA, detecting the single-stranded G-rich telomeric overhang. (c, d) The DNA was denatured within the gel and re-hybridized to the same probe to detect the entire telomeric DNA. Representative gels are shown. (e) The native signal for each sample was measured by *ImageQuant-TL*, and all the samples within each gel (a total of six gels) were normalized to the WT (M/M) sample and plotted (summarized in Supplementary Data 4). The slope for K/K was analyzed by simple linear regression. The equation for the regression line with 95% confidence intervals was Y= 0.0173*X + 1.13, and the P value corresponding to the deviation of the slope from zero is indicated above the graph. The high P value indicates no significant change in the overhang signal while telomeres shorten over seven generations of Telomouse. When comparing the K/K overhang signal to the M/M mice, the regression line (dotted line; equation: Y= 0.0074*X + 0.89) gave a P value of 0.50, indicating no significant difference in the overhang signal between the Telomouse and WT mouse.
