# Supplementary material for: Telomouse—a mouse model with human-length telomeres generated by a single amino acid change in RTEL1
Source: Nat Commun. 2023 Oct 23;14:6708. doi: 10.1038/s41467-023-42534-6 (PMC10593777; doi:10.1038/s41467-023-42534-6)
Supplement: Supplementary file 2 — Description of Additional Supplementary Files [file 41467_2023_42534_MOESM2_ESM.pdf]

Description of Additional Supplementary Files

Supplementary Data 1: TRF length and overhang - MEFs. 121 MEF samples (35 K/K from a generation three (F3) embryo, 26 M/K from an F3 embryo, and 60 M/M) were analyzed by pulse-field gel electrophoresis (PFGE) in nine independent gels (indicated by numbers). First, the native DNA was hybridized to a telomeric C-rich probe to detect the single-stranded G-rich telomeric overhang (OH). The native signals for 77 samples were measured by *ImageQuant-TL* in six gels and normalized to M/M PD10 or M/M PD18 as indicated. Then, the DNA was denatured in the gel and re-hybridized to the same probe. Mean TRF and SD TRF for all the samples were measured using *Telotool*. The raw images of all gels are included in Source Data File.

Supplementary Data 2: Nanopore telomere reads – telomere length and chromosomal mapping.

Genomic DNA, extracted from the MEF, mouse and human samples (as indicated for each tab), was sequenced by nanopore sequencing. Telomere reads were identified and analyzed by the *Telomere Analyzer* program. Listed are all qualified reads for each sample that are longer than the 'running' median (see Supplementary Data 3). The columns describe the following information for each read. Read ID: ID of each Nanopore read, as obtained from the Nanopore run. Each ID is unique; Read length: Length (nt) of the whole read as obtained from the Nanopore run, subtelomeric region included (if exists); Telomere start: Telomere start point (nt) within the Nanopore read; Telomere end: Telomere end point (nt) within the Nanopore read; Telomere length: Length of the telomere (nt) within the Nanopore read, equals to "Telomere_end" minus "Telomere_start"; Telomere density: Frequency of

telomeric repeat sequence (TTAGGG) within the telomere portion of the Nanopore read; Chromosome end: The chromosome end to which the read was mapped to. Head - P arm, Tail - q arm, NA - not assigned; Truncated telomere: An asterisk symbol is placed in cells where the Nanopore read does not contain a subtelomeric region. If the telomere start point was within the first 100 nt of the read, it was considered truncated.

Supplementary Data 3: Nanopore telomere reads – The influence of read length on median and average telomere length calculations.

Genomic DNA, extracted from the MEF, mouse and human samples (as indicated for each tab) was sequenced by nanopore sequencing. Telomere reads were identified and analyzed by the *Telomere Analyzer* program. The telomeric reads for each sample indicated in each tab were sorted based on their read length from largest to smallest. Then, 'running' median and 'running' average telomere length (TL) were calculated; for each telomere read we calculated the median and average TL of all reads of equal or greater length. Listed are all qualified reads for each sample. The columns describe the following information for each read. Read ID: ID of each Nanopore read, as obtained from the Nanopore run. Each ID is unique; Read length: Length (nt) of the whole read as obtained from the Nanopore run, subtelomeric region included (if exists); Telomere start: Telomere start point (nt) within the Nanopore read; Telomere end: Telomere end point (nt) within the Nanopore read; Telomere length: Length of the telomere (nt) within the Nanopore read, equals to "Telomere_end" minus "Telomere_start"; Telomere density: Frequency of telomeric repeat sequence (TTAGGG) within the telomere portion of the Nanopore read; RunAve_TL: The running average of the telomere lengths, after sorting the observations by read length, from longest to shortest; RunMed_TL: The running median of the telomere lengths, after sorting the observations by read length, from longest to shortest; ReadLen-RunMed_TL: The difference between the read length and the running median

telomere length, up to that point; RunMed_RL: The running median of the read lengths, after sorting the observations by read length, from longest to shortest; Truncated telomere: An asterisk symbol is placed in cells where the Nanopore read does not contain a subtelomeric region. If the telomere start point was within the first 100 nt of the read, it was considered truncated.

Supplementary Data 4: TRF length and overhang - Mice.

A total of 130 mice [106 K/K and 24 M/M (100% pure Blk6)] were analyzed multiple times by pulse-field gel electrophoresis (PFGE) in 12 gels for blood (119 samples) and 19 gels for tail (185 samples). The average age for K/K mice analyzed in the gels was 331 days, while the average age for M/M mice was 395 days. The native overhang (OH) signals measured in five (tail) and four (blood) gels were measured by *ImageQuant-TL* and normalized to the M/M samples highlighted in yellow for each gel. Mean TRF and SD TRF for all the samples were measured using *Telotool*. The raw images of all gels are included in Source Data File.
