# Supplementary material for: Telomouse—a mouse model with human-length telomeres generated by a single amino acid change in RTEL1
Source: Nat Commun. 2023 Oct 23;14:6708. doi: 10.1038/s41467-023-42534-6 (PMC10593777; doi:10.1038/s41467-023-42534-6)
Supplement: Supplementary file 7 — Reporting Summary [file 41467_2023_42534_MOESM7_ESM.pdf]

nature portfolio

Corresponding author(s): Yehuda Tzfati Ph.D. and Klaus H. Kaestner Ph.D.

Last updated by author(s): October 13, 2023

Reporting Summary

Nature Portfolio wishes to improve the reproducibility of the work that we publish. This form provides structure for consistency and transparency in reporting. For further information on Nature Portfolio policies, see our Editorial Policies and the Editorial Policy Checklist.

Statistics

For all statistical analyses, confirm that the following items are present in the figure legend, table legend, main text, or Methods section.

n/a	Confirmed	
☐	☒	The exact sample size (*n*) for each experimental group/condition, given as a discrete number and unit of measurement
☐	☒	A statement on whether measurements were taken from distinct samples or whether the same sample was measured repeatedly
☐	☒	The statistical test(s) used AND whether they are one- or two-sided<br>*Only common tests should be described solely by name; describe more complex techniques in the Methods section.*
☒	☐	A description of all covariates tested
☒	☐	A description of any assumptions or corrections, such as tests of normality and adjustment for multiple comparisons
☐	☒	A full description of the statistical parameters including central tendency (e.g. means) or other basic estimates (e.g. regression coefficient) AND variation (e.g. standard deviation) or associated estimates of uncertainty (e.g. confidence intervals)
☐	☒	For null hypothesis testing, the test statistic (e.g. *F*, *t*, *r*) with confidence intervals, effect sizes, degrees of freedom and *P* value noted<br>*Give P values as exact values whenever suitable.*
☒	☐	For Bayesian analysis, information on the choice of priors and Markov chain Monte Carlo settings
☒	☐	For hierarchical and complex designs, identification of the appropriate level for tests and full reporting of outcomes
☒	☐	Estimates of effect sizes (e.g. Cohen's *d*, Pearson's *r*), indicating how they were calculated

Our web collection on statistics for biologists contains articles on many of the points above.

Software and code

Policy information about availability of computer code

Data collection	Nanopore MinKNOW application<br>Nanopore Guppy application
Data analysis	Phyre 2.0 - Protein Homology/analogY Recognition Engine<br>Telotool program<br>ImageJ<br>Image Quant<br>Telometer<br>GraphPad Prism 8.0<br>Nanopore MinKNOW application<br>Nanopore Guppy application<br>minimap2<br>https://github.com/Tzfatilab/Telomere-Analyzer - The scripts developed in this work for processing Nanopore sequencing reads

For manuscripts utilizing custom algorithms or software that are central to the research but not yet described in published literature, software must be made available to editors and reviewers. We strongly encourage code deposition in a community repository (e.g. GitHub). See the Nature Portfolio guidelines for submitting code & software for further information.

Data

Policy information about availability of data

All manuscripts must include a data availability statement. This statement should provide the following information, where applicable:

- Accession codes, unique identifiers, or web links for publicly available datasets
- A description of any restrictions on data availability
- For clinical datasets or third party data, please ensure that the statement adheres to our policy

All data generated or analyzed during this study are included in this published article, its supplementary information files, and the public repository OSF.

Representative gels are shown in Figures 2, 7, S2-S4, and S9-S12. All measured and calculated values are provided in Supplementary Data files 1 (MEFs) and 4 (mice). The complete un-spliced gel images for Figure 2a are shown in Figure S2a,c,e. The complete un-spliced gel images for Figure 7b, including the native hybridization, are shown in Figure S11d,h. The original gel images are included in the Source Data file.

The Nanopore telomeric read characteristics are summarized in Supplementary Data files 2 and 3. The raw sequencing data are available on the public repository OSF.

Any additional data are available upon request to the corresponding authors.

Research involving human participants, their data, or biological material

Policy information about studies with human participants or human data. See also policy information about sex, gender (identity/presentation), and sexual orientation and race, ethnicity and racism.

Reporting on sex and gender	N/A
Reporting on race, ethnicity, or other socially relevant groupings	N/A
Population characteristics	N/A
Recruitment	N/A
Ethics oversight	N/A

Note that full information on the approval of the study protocol must also be provided in the manuscript.

Field-specific reporting

Please select the one below that is the best fit for your research. If you are not sure, read the appropriate sections before making your selection.

☒ Life sciences ☐ Behavioural & social sciences ☐ Ecological, evolutionary & environmental sciences

For a reference copy of the document with all sections, see nature.com/documents/nr-reporting-summary-flat.pdf

Life sciences study design

All studies must disclose on these points even when the disclosure is negative.

Sample size	Sample size was dictated by the number of MEFs and mice available, and by the nanopore sequencing yield. Statistical tests confirmed the significance of the results with the available sample sizes.
Data exclusions	One wild type (M/M) MEF culture was excluded because it was obtained by crossing two heterozygous RTEL1 mutants, and thus inherited short telomeres (M/M** in supplementary figure 2).<br>Three samples were excluded from the gels shown (M/M PD 160 and M/M PD 250 in supplementary figure 2 and M/M PD 70 in supplementary figure 3) because they were suspected to be degraded. These samples were repeated and the correct results were included in the analysis.
Replication	All attempts at samples replication (summarized in figure legends and supplementary Tables) were successful.
Randomization	Animals were randomized to treatment to avoid confounders.<br>Samples from each mouse generation were allocated randomly to different gels with at least one wild type control sample was included in each gel. MEF samples were arranged by PD order and wild type control sample(s) were included in each gel.<br>Immunoflourescence images were taken randomly.<br>FISH images for quantifying aberrations, CO-FISH, and meta-TIF were taken randomly.<br>Images for human and mouse pairs were taken randomly.

Blinding	Blinding was done whenever possible, e.g., processing gel images, microscopic images and nanopore sequencing data.

Reporting for specific materials, systems and methods

We require information from authors about some types of materials, experimental systems and methods used in many studies. Here, indicate whether each material, system or method listed is relevant to your study. If you are not sure if a list item applies to your research, read the appropriate section before selecting a response.

Materials & experimental systems

n/a	Involved in the study
☐	☒ Antibodies
☐	☒ Eukaryotic cell lines
☒	☐ Palaeontology and archaeology
☐	☒ Animals and other organisms
☒	☐ Clinical data
☒	☐ Dual use research of concern
☒	☐ Plants

Methods

n/a	Involved in the study
☒	☐ ChIP-seq
☒	☐ Flow cytometry
☒	☐ MRI-based neuroimaging

Antibodies

Antibodies used

Primary antibodies used were:
Mouse monoclonal TRF1 antibody (Abcam; AB10579)
Rabbit polyclonal γH2AX (Cell Signaling Technology; 2577S)
Mouse monoclonal γH2AX (Millipore, JBW 301)
Mouse anti-E-Cadherin (610181, 1:500, BD Biosciences)
Rabbit polyclonal anti-FAH (ThermoFisher; PA542049)
Goat polyclonal anti-GFP (Abcam; AB6673)
Secondary antibodies:
Cy2 AlexaFluor 488 Goat anti Mouse IgG (ThermoFisher #A-11029).
Cy3 AlexaFluor 594 Donkey anti Rabbit IgG (ThermoFisher #A-21207)
TSA-conjugated secondary antibodies (Cy2 and Cy3)
Cy2 anti-mouse secondary antibody.
TSA-conjugated secondary antibodies (Cy2 and Cy3) were used.

Validation

TRF1 (Abcam; AB10579) - Validation statement by manufacturer: "Validated in WB, ICC/IF and tested in Human samples." https://www.abcam.com/en-hr/products/primary-antibodies/anti-trf2-trf1-antibody-trf-78-ab10579

γH2AX (Cell Signaling Technology) - "we validate all CST® antibodies, assay kits, and reagents to ensure optimal performance in the approved applications shown on our product web pages." - https://www.cellsignal.com/about-us/our-approach-process/cst-antibody-performance-guarantee

γH2AX (Millipore, JBW 301) - "Evaluated by Western Blotting in HeLa cell lysate." - https://www.merckmillipore.com/INTL/en/product/Anti-phospho-Histone-H2A.X-Ser139-Antibody-clone-JBW301,MM_NF-05-636-I?ReferrerURL=https%3A%2F%2Fwww.google.com%2F

Eukaryotic cell lines

Policy information about cell lines and Sex and Gender in Research

Cell line source(s)	All mouse embryonic fibroblast cell lines were established and immortalized by us.
Authentication	Mutations were validated by PCR and sequencing.
Mycoplasma contamination	The cell lines were tested for mycoplasma and found negative
Commonly misidentified lines (See ICLAC register)	No commonly misidentified cell lines were used in the study.

Animals and other research organisms

Policy information about studies involving animals; ARRIVE guidelines recommended for reporting animal research, and Sex and Gender in Research

Laboratory animals	Mus musculus, C57BL/6J and Mus musculus, C57BL/6J Rtel1 mutant mice. Mice ages are mentioned in detail in the "Life Sciences Study Design" section of this reporting summary.
Wild animals	No wild animals were used in the study.
Reporting on sex	Both male and female mice were used with comparable results.
Field-collected samples	No field collected samples were used in the study.
Ethics oversight	The use of animals in this study followed the Guide for the Care and Use of Laboratory Animals, Laboratory Animal Ordinances, and the Animal Welfare Act. The study was approved by the University of Pennsylvania Institutional Animal Care and Use Committee, protocol number 805623.

Note that full information on the approval of the study protocol must also be provided in the manuscript.
